# Editorial: Undetermined implications of chronutrition: a missing curriculum in medicine

**DOI:** 10.3389/fnut.2023.1290017

**Published:** 2023-09-29

**Authors:** Reza Rastmanesh, Adviye Gulcin Sagdicoglu Celep, Abraham Wall-Medrano

**Affiliations:** ^1^American Physical Society, College Park, MD, United States; ^2^Faculty of Health Sciences, Nutrition and Dietetics Department, Gazi University, Ankara, Türkiye; ^3^Coordinación de Ciencias Químico-Biológicas, Instituto de Ciencias Biomédicas, Universidad Autónoma de Ciudad Juárez, Ciudad Juárez, Chihuahua, Mexico

**Keywords:** chronobiology, chrononutrition, sleep, chronic diseases, zeitgeber

Chronobiology (a.k.a. circadian biology) studies the rhythmicity of physiological processes throughout a 24-h cycle in any living organism. The body's timekeeping system is composed of a central (suprachiasmatic nucleus) clock and many peripheral (organ-specific) clocks, responsible for aligning body functions to timing photic (light/darkness) cues and non-photic (food intake) external cues called zeitgebers (1, 2). Even though chronobiology dates back to the Hippocratic era, its recognition as a scientific biomedical discipline began recently with the pioneering work conducted by Jeffrey Hall, Michael Rosbash, and Michael Young on the molecular mechanisms that govern circadian rhythmicity, research that earned them the Nobel Prize in 2017 (3). It is noteworthy that the circadian rhythm is controlled by transcriptional–translational feedback loops of clock genes and proteins that further interact with a well-orchestrated neuroendocrine system, connecting both central and peripheral clocks (4). However, the empirical observation of an “appetite-satiety” circadian rhythm that governs the body's energy balance, sleep, cardio-metabolic function, and many other nutrition-sensitive metabolic processes gave rise to chrononutrition as a scientific discipline. Chrono-disruptive eating behaviors have been implicated in many health disorders including sleep disturbances, cardiometabolic risk, unbalanced energy mobilization, body temperature deregulation, weight gain, psychosocial distress, and redox imbalances (3–5). Conversely, a substantial body of evidence indicates that meal timing, diet quality, and the regular intake of key dietary chronobiotics (e.g., phytomelatonin, tryptophane, short-chain fatty acids, and retinoic acid) help to synchronize internal clocks, reinforcing the idea that long-term dietary interventions with these food bioactives may be effective on restoring the body's circadian rhythmicity and metabolic homeostasis (5, 6).

In this special topic of *Frontiers in Nutrition*, new evidence has been gathered on the state of the art of chrononutrition and its implications. While remaining an emerging discipline in the biomedical curriculum, these studies offer valuable information in the form of narrative/systematic reviews, epidemiological studies, preclinical evidence, and clinical studies in this field. In their narrative review, Gangitano et al. documented the role of specific nutrient intake (chrono-disruptors or positive regulators) in the structure and quality of sleeping patterns as a bidirectional phenomenon related to obesity and glycemic abnormalities. The authors particularly emphasize the role of the hormonal circuit that connects to this bidirectional axis (evidence from preclinical studies) and the beneficial effect of specific nutrients [e.g., tryptophan (Trp): large neutral amino acids (LNAA) ratio] and the negligible effectiveness of intermittent fasting (IF) protocols (clinical evidence). Regarding the latter, He et al. reported the short-term immunomodulatory, anti-obesogenic, and biochemical effects of IF protocols (mostly randomized-controlled trials), concluding that IF protocols induce modest immunomodulatory effects in healthy people and those with special physiological (e.g., pregnancy) and underlying pathophysiological (e.g., obese) conditions, but that such effects come from different mechanisms. Lastly, Qu documented, succinctly yet in-depth, the molecular crosstalk between the circadian clock and cancer development (e.g., oncogene primordial targeting)/progression (e.g., chromatin remodeling and angiogenesis) and its therapeutic implications, explicitly reviewing the effectiveness of IF and chrono-chemotherapy in cancer.

Epidemiological studies represent the first step when searching for scientific evidence linking the circadian rhythm with the health-disease continuum. In observational studies involving adults, it has been stated that night-shifting, evening chronotypes, and late sleep or meal timing are strong chrono disruptors that may lead to weight gain, hypertension/dyslipidemia, chronic inflammation, and ultimately cardiometabolic diseases and type-2-diabetes (7). However, little is known about the impact of chronodisruption in young or metabolically compromised populations. Juliana et al. studied the chrononutrition behavior of 409 young (21.5 ± 2.2 y old). Malayan participants during the COVID-19 pandemic, observing similar eating patterns to other young populations, variable sleep patterns, and a scarce yet significant chrono-disruptive eating pattern in participants with low body weight. Kuwahara et al. aimed to document the causal association of breakfast styles (Japanese, Western, and cereal consumers) with sleeping/eating patterns and lifestyle factors in Japanese children (aged 3–8 years), observing that those eating Japanese style breakfast > Western style breakfast and morning > evening type have better sleep and eating patterns. On the other hand, Teoh et al. followed 20 healthy primigravids during the second and third trimesters of pregnancy, documenting their eating patterns/style (3-day food records) and melatonin and cortisol levels (saliva, 5-time points in 24 h) during both periods; the authors' findings suggested that certain chrononutrition-related behaviors (e.g., eating window and breakfast skipping) have a significant influence on maternal melatonin and cortisol rhythmicity and so, targeting these intake behaviors may help to restore the circadian rhythm of melatonin and cortisol.

In the second and third stages of epidemiological research, the molecular/physiological mechanisms and benefits of chrononutrition interventions are commonly documented; this Research Topic includes two experimental studies using murine models and three clinical and community interventions that offer evidence in this regard. Zou et al. aimed to analyze the transcriptomic/metabolomic alterations in the rhythmic transcriptome and metabolism of meibomian glands (MGs; eyelid sebaceous glands) of C57BL/6J mice fed a balanced diet or a high-fat diet (HFD) and maintained in a 12/12 h dark/light cycle. The authors observed that HFD induces a chrono-disrupted state characterized by altered rhythmic oscillations of lipid components (enriched signaling pathways: glycerolipid/glycerophospholipid/ether metabolism, lipid storage deviations) but that HFD did not induce desynchrony of the light-regulated central clock pacemaker. Trebucq et al. induced a transient 12/12 h light/dark (LD) desynchronization state mimicking a chronic jet lag (CJL) condition to evaluate the ultimate effects on daily energetic homeostasis and weight gain, observing that this chronic misalignment causes glycemic abnormalities (nocturnal hyperglycemia, glucose intolerance, and hyperinsulinemia), high LDL, and weight gain. As for interventions with humans, Jacob et al. evaluated the eating behaviors/timing and certain psychological traits in 301 overweight/obese middle-aged participants (56% women) in weight loss programs, documenting that late eating is associated with a higher total energy intake and suboptimal eating behaviors, worsening factors associated with a higher risk of obesity. Marciniak et al. evaluated the plausible causal relationship between sex, chronotype, and age with saliva cortisol and dehydroepiandrosterone (DHEA) 64 h-rhythmicity after 1-day fasting in 49 obese individuals (50% women) observing mainly sex and chronotype-specific phasing alterations. Lastly, Albreiki et al. investigated the possible effect of melatonin supplementation on the transient fluctuation of plasma leptin and subjective appetite rating in nine young male eutrophic participants in a randomized three-way (two light exposures, one melatonin supplementation) cross-over overnight (6 PM-6 AM) study. The authors documented a positive impact of exogenous melatonin on subjective hunger and desire to eat and plasma leptin levels, despite a greater efficacy using MT1/MT2 receptor agonists to achieve appetite control.

## Author contributions

RR: Conceptualization, Formal analysis, Investigation, Project administration, Supervision, Writing—review and editing. AG: Formal analysis, Supervision, Validation, Writing—review and editing. AW-M: Conceptualization, Formal analysis, Investigation, Project administration, Supervision, Writing—original draft, Writing—review and editing. All authors contributed to the article and approved the submitted version.
